# Aging in Spaceflight, Oxidative Stress, and Microbiome Dysbiosis: A Comprehensive Review

**DOI:** 10.3390/ijms27177644

**Published:** 2026-08-26

**Authors:** Charles C. Naney, Joseph L. Graves, Scott H. Harrison

**Affiliations:** Department of Biology, North Carolina Agricultural and Technical State University, Greensboro, NC 27411, USA; cnaney2@gmail.com (C.C.N.); gravesjl@ncat.edu (J.L.G.J.)

**Keywords:** aging, microbiota, oxidative stress, spaceflight

## Abstract

The evolutionary theory of aging demonstrates that the force of natural selection declines with age in multicellular organisms. Two population genetic mechanisms are consistent with the evolutionary theory: mutation accumulation and antagonistic pleiotropy. These in turn account for the physiological, cellular, and molecular mechanisms associated with aging. Spaceflight provides an opportunity to examine how organisms physiologically acclimate to environmental conditions. Mitochondria and the gut microbiome are central regulators of host metabolism, redox homeostasis, immune function, and physiological resilience, and both are impacted by host adaptations and acclimations. Mitochondrial dysfunction and oxidative stress have consequently emerged as important candidate mechanisms linking biological aging and spaceflight-associated physiological change. This review examines evidence surrounding mitochondrial dysfunction, oxidative stress, and gut microbiome dysbiosis across humans, mice, and fruit flies under spaceflight and corresponding terrestrial control conditions. This review reports further on the progression of theory and recent evidence from spaceflight missions for how biomarker genes most associated with mutation accumulation and antagonistic pleiotropy appear to have a core role in natural aging and extreme physiological acclimation.

## 1. Introduction

The modern study of aging biology now integrates evolutionary theory, population genetics, physiology (metabolic regulation), and cellular and molecular biology to investigate functional decline across organisms [1,2,3]. Subsequent systems-level analyses have examined how spaceflight perturbs cellular mechanotransduction, producing aging-associated signs of change in the extracellular matrix, nuclear lamina, and the linker of the nucleoskeleton (LINC) complexes [4]. Spaceflight is not a single experimental treatment, but a combination of distinct physical and environmental exposures. While ground-based approaches aim to simulate microgravity conditions, this has yet to be proven in most cases. Consequently, biological responses should be interpreted within the experimentally defined boundary conditions under which they were observed.

Spaceflight conditions include a period of microgravity in space along with a combination of launch- and return-associated hypergravity. In addition, these conditions further include ionizing radiation, confinement, and conditions that may be specific to the utilized spacecraft. Analyses of organismal response to spaceflight can be based on primary data from new experiments performed in actual missions or secondary re-analyses and integration of existing mission datasets using multi-omics and other computational systems biology approaches. In contrast, ground-based analogs are a much larger continuum of simulated spaceflight approaches that may only emulate a subset of exposure factors depending on their control environments, and experimental protocols specifying simulated spaceflight exposure using clinostats, random positioning machines, hindlimb unloading, bed rest, and dry immersion [4]. Terrestrial-based studies (aging without spaceflight) provide a comparative basis for understanding these other studies, and include observations derived from basal, vivarium, and ground control groups as included in the NASA GeneLab Open Science Data Repository (OSDR) [5]. Table 1 summarizes the extent to which these different categories of investigations for spaceflight, secondary studies, ground-based analog studies, and terrestrial studies are represented in this review. Specific literature articles are as indicated in the Supplemental Materials (Table S1).

Advancing this concept further, the proposed signs of aging framework establishes a mechanistic foundation for organizing spaceflight-related observations across cellular regions related to DNA and epigenetic regulation, mitochondrial function, and other aspects of aging physiology. Other recent investigations have suggested that prolonged spaceflight exposure may induce physiological changes overlapping multiple hallmarks of aging, including mitochondrial dysfunction, oxidative stress accumulation, immune dysregulation, inflammation, genomic instability, and altered intercellular communication [6,7,8,9,10]. Mitochondria have emerged as central regulators of cellular energetics, redox homeostasis, and stress-response pathways, and are recognized as having a primary role underlying the physiological effects of spaceflight. There is growing evidence for how exposure to microgravity, ionizing radiation, and associated metabolic stressors may perturb oxidative phosphorylation, mitochondrial maintenance, and cellular homeostasis across tissues and organ systems [11].

Mitochondria are inherent to multicellular life, including almost all Metazoans [12]. Originating from an endosymbiotic event that likely occurred only once in life’s diversification, mitochondria are central regulators of cellular energetics and metabolism in many eukaryotic organisms [13]. Depending on tissue type and metabolic demand, most animal cells contain hundreds to thousands of mitochondria, with notable exceptions such as mature red blood cells, which lack mitochondria entirely [14]. Through oxidative phosphorylation (OXPHOS), mitochondria generate adenosine triphosphate (ATP) and participate in numerous metabolic pathways, including the citrate cycle, fatty acid oxidation, glutamine metabolism, and gluconeogenesis.

Interest in mitochondria as a mechanistic explanation for aging increased following the development of the free radical theory of aging by Denham Harman in the 1950s. Harman proposed that reactive oxygen species (ROS) generated during cellular respiration contribute to progressive oxidative damage associated with physiological aging [15]. A proposed general role of mitochondrial free radicals with aging has further linked mitochondrial dysfunction, oxidative stress accumulation, and impaired cellular maintenance to senescence and age-related decline [16,17]. Physiological changes associated with prolonged spaceflight have likewise been correlated with mitochondrial dysfunction and oxidative stress responses, suggesting that some mechanisms implicated in terrestrial aging may also contribute to spaceflight-associated physiological decline [11]. It has been more generally proposed that antagonistic pleiotropy accounts for not only increased mitochondrial dysfunction but other drivers of aging such as protein misfolding, telomere attrition, and DNA damage [18]. For how spaceflight may accelerate senescence, attention is being increasingly given to how not only mitochondrial dysfunction but other outcomes of genomic instability and telomere attrition are likewise affected for humans exposed to spaceflight [19].

Recent systems-level studies have utilized transcriptomic and multi-omics investigations to identify alterations in mitochondrial and oxidative-stress-associated pathways during both aging and spaceflight exposure. However, the directionality of these responses remains heterogeneous across studies, with both upregulation and downregulation reported depending on species, tissue type, exposure duration, and experimental conditions [6,11,20,21,22,23,24]. Beyond mitochondrial signaling, spaceflight has also been associated with host–microbiome dysregulation, altered inflammatory responses, and shifts in microbial community composition across model organisms and human cohorts [25,26,27,28,29,30,31,32]. Collectively, these findings have led to development of countermeasures targeting oxidative stress, immune dysregulation, radiation-associated damage, and mitochondrial dysfunction associated with prolonged spaceflight exposure [33,34,35,36,37,38,39,40,41,42,43,44,45,46,47]. There is at present a wealth of scientific reports on aging that have been achieved across different species, spaceflight and earth-based conditions, physiologic evaluations, and biotechnological platforms (Table 1).

## 2. Metabolism and Aging: Oxidative Stress Accumulates

Amino acid metabolism is an important source of both energy and stability for cellular function [48]. Metabolic pathways for most amino acids intersect within mitochondrial processes, highlighting the central role of mitochondria in amino acid metabolism and cellular energetics [49]. There are over 200 known mitochondrial metabolic reactions involved in cellular energetics, biosynthesis, and physiological maintenance [50]. The tricarboxylic acid (TCA) cycle is a critical series of redox metabolic reactions within the mitochondria that couple environmental energy to cellular respiration. During oxidative phosphorylation, mitochondria generate reactive oxygen species (ROS) as byproducts of electron transport, while nitrogen species (RNS) arise through interactions between nitric oxide and cellular redox pathways. Additional endogenous sources of reactive species may be generated through inflammatory responses and immune activity. Under normal physiological conditions, antioxidant systems regulate reactive oxygen and nitrogen species (RONS) production and maintain redox balance; however, disruption of these systems may result in oxidative stress through accumulation of reactive intermediates and associated molecular damage [51]. Oxidative stress may be further amplified by environmental exposures relevant to spaceflight, including ionizing radiation, hypoxia, and toxic compounds, which contribute to mitochondrial dysfunction, lipid peroxidation, protein oxidation, and DNA damage [20]. Reactive species implicated in these processes include nitric oxide (•NO), superoxide (•O_2_^−^), hydrogen peroxide (H_2_O_2_), and mitochondrial-derived reactive oxygen species generated during oxidative phosphorylation and electron transport chain leakage [52].

Given the central role of mitochondria in cellular energetics, signaling, and physiological maintenance, it is not surprising that mitochondrial dysfunction has been implicated in numerous pathological conditions, including cancer, cardiovascular disease, Type II Diabetes, neurodegenerative disorders, as well as cellular senescence associated with biological aging. A major component of mitochondrial dysfunction involves disruption of redox homeostasis associated with increased RONS production and accumulation [53]. Under normal conditions, antioxidant and quality-control systems regulate these reactive intermediates; however, persistent oxidative stress may result in oxidative damage to proteins, lipids, mitochondria, and DNA. Cells experiencing sustained oxidative damage may activate stress-response pathways leading to altered cellular function, senescence, apoptosis, or necrotic tissue injury depending on the severity and duration of the insult. These mechanisms are increasingly relevant in spaceflight biology, where ionizing radiation, microgravity, hypoxia-related signaling, immune alterations, and metabolic stress may further perturb mitochondrial function and redox homeostasis. The strain on mitochondrial networks by the accumulation of mtDNA mutations in patients with the HTT mutation results in accumulation of oxidative stress in later ages from the leakage of ROS from malformed mitochondrial proteins that is not present in younger patients [54], an often-cited example of antagonistic pleiotropy. Mutation accumulation and antagonistic pleiotropy mechanisms have been generally shown to control life span via experimental evolution studies in yeast, round worms, fruit flies, and mice [55,56,57,58], and population genetic mechanisms may overall be considered to engender various physiological, cellular, and molecular mechanisms of aging [59,60].

In healthy cells, mitochondria maintain cellular homeostasis through tightly regulated redox reactions that support oxidative phosphorylation (OXPHOS), including ATP production, and redox-dependent signaling pathways [61,62]. Foundational work by Peter Mitchell and Jennifer Moyle established the chemiosmotic coupling theory, demonstrating how electron transport and proton gradients drive mitochondrial bioenergetics and ATP synthesis through oxidative phosphorylation [63]. Under normal physiological conditions, antioxidant systems regulate reactive oxygen species and maintain redox balance; however, disruption of these regulatory systems may contribute to oxidative stress and altered cellular function [64]. Recent work in systems biology and evolutionary metabolism has further emphasized the complexity of mitochondrial metabolic networks associated with longevity and aging across species, including *Drosophila* models [65]. As these multivariable interactions span redox metabolism, signaling, and gene regulation, and physiological state changes, computational and machine-learning approaches are increasingly relevant for modeling complex aging and spaceflight-associated biological systems [66].

Accumulation of oxidative stress has been widely implicated in aging, mitochondrial dysfunction, and cellular damage across multiple biological systems [4,10,17,51,67,68,69,70,71,72]. Oxidative stress arises through disruption of redox homeostasis and accumulation of reactive oxygen and nitrogen species (RONS) generated during metabolism, oxidative phosphorylation, immune signaling, and other bioenergetic processes central to cellular physiology [73,74,75]. Persistent oxidative stress may damage proteins, lipids, DNA, and adjacent mitochondria, contributing to physiological dysfunction, inflammation, immune dysregulation, and age-associated disease phenotypes [76,77,78].

Both biological aging and spaceflight exposure may further amplify oxidative stress through disruption of oxidative phosphorylation and increased accumulation of mitochondrial-derived reactive oxygen and nitrogen species (RONS). Electron leakage associated with respiratory chain complexes I–III is a major source of mitochondrial-derived reactive oxygen species [79]. In spaceflight environments, exposure to ionizing radiation, altered gravity, and associated metabolic stressors may further increase RONS accumulation and oxidative damage [80]. Long-duration spaceflight has additionally been associated with sustained inflammatory responses, immune dysregulation, and altered redox signaling in astronaut and cosmonaut cohorts [6,81]. Collectively, these findings support oxidative stress as a conserved physiological process associated with mitochondrial dysfunction, aging, and spaceflight-related cellular perturbation.

## 3. Oxidative Stress and Gut Microbiome Dysbiosis

As may ensue from both natural aging and environmental exposures, oxidative stress accumulates from mitochondrial dysfunction within host tissues and is also influenced by host–microbiome interactions that regulate metabolism, inflammation, and redox homeostasis. ROS and oxidative stress are key features of cardiovascular disease [82]. Mitigating oxidative stress has emerged as a potential strategy for reducing physiological risks associated with long-duration spaceflight. In addition, NASA researchers have already begun to associate how oxidative stress is involved in the human aging process. Takahashi et al. reported elevated cardiovascular disease mortality among Apollo astronauts approximately 40 years after lunar missions compared with astronauts who remained in Low Earth Orbit (LEO), suggesting possible long-term effects of deep-space radiation exposure and oxidative stress [20]. Targeted studies of oxidative stress are often centered upon mitochondrial-derived reactive oxygen and nitrogen species (RONS) and associated inflammatory signaling networks, but newer studies are implicating the microbiome. Many inflammatory, metabolic, and neuroendocrine signaling pathways are influenced by bidirectional communication along the gut–brain axis. The highest concentration of microbes in the human body is in the gut. Oxidative stress physiology is now being closely linked to host–microbiome interactions, particularly in cases where gut microbiome dysbiosis and accelerated aging are present [83]. Because gut microorganisms contribute to metabolism, immune regulation, inflammatory signaling, and redox balance, disruption of host–microbiome homeostasis may further amplify oxidative stress-associated physiological decline. Overall, studies conducted in both Earth-based and spaceflight environments have increasingly implicated microbiome dysbiosis in altered host physiology, inflammation, immune dysfunction, and metabolic perturbation across model organisms and human cohorts [6,84,85,86,87,88]. Because microbial metabolites influence mitochondrial metabolism, immune signaling, and redox homeostasis, dysbiosis may contribute to physiological changes associated with both aging and spaceflight.

Earth-based rodent studies have demonstrated that gut microbiome composition may influence mitochondrial physiology, oxidative stress responses, and recovery from tissue injury. For example, experimental microbiome studies in post-stroke rats identified protective physiological effects associated with specific microbial community structures compared to controls [89]. Metagenomic sequencing of murine fecal samples has further revealed microbiome-associated biosignatures linked to resistance or susceptibility to oxidative stress-related metabolic conditions, including differences observed between high-fat and lean cohorts [90]. These findings support the view that host–microbiome interactions contribute to metabolic regulation, mitochondrial function, and immune activity within gastrointestinal tissues that are highly enriched in mitochondria and immunologically active cell populations [87]. Microbial metabolites that can influence mitochondrial metabolism, immune signaling, and redox homeostasis through microbiome composition may contribute to host physiological resilience or susceptibility under both aging and spaceflight-associated conditions. Host–microbiome interactions are influenced by numerous biological and environmental variables, experimental studies increasingly consider variables including species, strain, breeding history, sex, age, conserved orthologous pathways, and disease phenotypes when evaluating microbiome-associated physiological responses across model systems [25,91,92,93].

Metabolites enter and exit Metazoan hosts through the gut, where the microbiome is particularly abundant and diverse in humans, mice, and fruit flies. An initial indicator of microbiome involvement in aging is the decline in gut microbial diversity frequently observed after approximately 65 years of age, with the trend intensifying after 80 years. Using the Kendall Uniqueness score (KUS), Bradley and Haran quantified associations between gut microbiome composition and healthy aging across populations in Europe, North and South America, and Asia. Their analysis identified distinct microbial taxa associated with healthy and unhealthy aging phenotypes. Unhealthy aging and gut microbiome dysbiosis were positively correlated with *Klebsiella*, *Ruminococcus*, and *Clostridium* genera. Inflammation specific to aging and changes in microbiome were associated with pathogens from *Klebsiella* and *Escherichia*. In contrast, healthy aging was associated with higher abundance of *Coprococcus*, *Akkermansia*, and *Faecalibacterium* genera. The bacterial species *F. prausnitzii* has exhibited specific anti-inflammatory effects [94].

All cells, host and microbial, are equipped with an ability to engage in metabolite sensing, and this is why a Molecules-mediated Bidirectional Interactions (MBIs) framework is useful for understanding and potentially controlling the interactome between the mitochondria, metabolism, and microbiome. The MBI framework advances this review by providing a common mechanistic intersection through which host cells and microbes can be evaluated as interconnected metabolic systems linked by shared metabolites, molecular signals, and redox-active compounds. This framework contrasts with studies that may otherwise evaluate fixed signals or other conditions leading to specific host cell or microbial responses within the system. This MBI framework can be arrived at by identifying a common core molecular metabolism between many bacteria and eukaryotic cells with common pathways including glycolysis and the TCA cycle, and with pathway byproducts and free radicals also likely encompassing similar ranges. RONS leakage may be anticipated to modulate not only clusters of adjacent host cells within an aging host, but also nearby bacteria [95]. Amino acid metabolism is particularly relevant because many amino acid degradation pathways intersect with the tricarboxylic acid (TCA) cycle, linking nutrient availability to mitochondrial function and cellular bioenergetics [48]. The TCA cycle serves a central role in both catabolic and anabolic metabolism and contributes to ATP production, biosynthesis, and redox homeostasis. Recent rodent studies have further suggested that alterations in amino acid metabolism may contribute to disruption of TCA cycle activity [96]. In spaceflight environments, nutritional strategies that support mitochondrial function and limit excessive oxidative stress may therefore represent an important area for future countermeasure development [97].

Growing evidence of oxidative stress accumulation in spaceflight in host physiology of astronauts and model organisms continues to be collected. Several spaceflight-associated conditions have been proposed to contribute to gastrointestinal dysfunction, including microbiome alterations associated with changes in microbial metabolite production, such as propionate and phenolic acids linked to Bacteroides populations, along with decline of *F. prausnitzii* [98]. Conversely, *F. prausnitzii* appears to be one of the most abundant bacteria in a healthy gut microbiome and has been associated with anti-inflammatory physiological effects [99]. Data returned from extended missions aboard the International Space Station (ISS) have documented gastrointestinal distress, respiratory infections, and skin disorders, all of which involve tissues characterized by extensive host–microbiome interaction [100]. These observations suggest that alterations in microbiome composition may influence inflammatory signaling, metabolic homeostasis, and oxidative stress associated with physiological responses. Glucose is one of multiple classes of metabolites converted by microbiota in the gut, illustrating how microbial metabolism may influence host energetic pathways, immune function, and overall physiological homeostasis [101].

Research conducted aboard the ISS, including the NASA Twins Study and parallel investigations in murine models, has substantially expanded understanding of the physiological consequences of spaceflight. These studies have revealed coordinated changes involving mitochondrial dysfunction, oxidative stress, metabolic dysregulation, microbiome dysbiosis, immune dysregulation, and inflammation across multiple biological systems [33,102,103,104]. Importantly, many of these physiological findings are not limited to spaceflight. Studies of natural aging on Earth in humans greater than 65 years and C57BL/6 variant mice over 20 months have similarly suggested that microbiome-derived metabolites influence host immune cell function and host physiology [105]. Likewise, transcriptomic analyses of microglia from aging mouse brains have demonstrated increased ROS accumulation with its association with metabolic shift and excitation of microglia [106]. Collectively, these findings support the view that interactions among oxidative stress, metabolism, immunity, and host–microbiome physiology may represent conserved features of both biological aging and spaceflight-associated physiological changes.

We consider studies that evaluate strategies for mitigating mitochondrial dysfunction and oxidative stress associated with aging on Earth and during spaceflight. Attention is given to interventions that examine reciprocal interactions between microbes and host cells through MBIs [95]. These interactions may influence reactive oxygen and nitrogen species (RONS), inflammatory signaling pathways, microbial phenotypes, and physiological processes associated with aging-related dysfunction and degenerative disease. Experimental evidence further suggests that specific metabolites and hormonal pathways may directly influence microbial growth and virulence. For example, growth of *Helicobacter pylori* has been reported to be inhibited by somatostatin and promoted by gastrin, while virulence-associated activity in *Burkholderia pseudomallei* and *Burkholderia cepacia* may be enhanced by insulin signaling [95]. Related concepts involving metabolite-dependent host–microbe communication have also been proposed through broader microbe–host signaling models linking metabolism, immunity, and microbial ecology [107].

There is likely an even deeper range of metabolic dynamics that may ultimately impact gut microbiome dysbiosis. These include bone demineralization, muscle atrophy, liver atrophy, cognitive functional decline, cardiovascular disease, macular decay, endocrine disorders, respiratory infections, and even cell death [6,11,108,109]. Since these outcomes emerge across multiple tissues and physiological systems, longitudinal measurements and temporal case data are essential for understanding their progression. Within this context, oxidative stress represents a particularly informative physiological process because it intersects with mitochondrial function, metabolism, immune regulation and host–microbiome interactions, all of which have been implicated in both biological aging and spaceflight.

Spaceflight and biological aging may alter redox metabolic processes associated with oxidative stress and cellular stress-response pathways, including those regulated by p53 which has a coordinating role between oxidative stress, response to DNA damage, and apoptotic pathways [72]. This is because maintenance of redox homeostasis is essential for cellular metabolism, signaling, and tissue integrity across the major organs. Distribution of oxidative stress may then have consequences across multiple organ systems. Persistent oxidative stress may become a driver of oxidative damage to proteins, lipids, and DNA thereby becoming a systematic insult to cellular function and physiological resilience, especially when reactive oxygen and nitrogen species (RONS) accumulate beyond the capacity of antioxidant defense systems [11,23,68]. In addition, oxidative stress can damage the internal cell environment sufficiently to stop the cell cycle. This includes the accumulation of DNA damage and mutations in addition to degradation of other cellular components that can lead to cellular senescence or other outcomes such as apoptotic pathway [110]. Even more recent studies have indicated that increased oxidative stress in host gut tissues may favor growth of pathogenic or proinflammatory bacteria populations, further highlighting potential interactions between host redox homeostasis and microbiome composition [78]. Work has been progressing toward identifying specific biomarkers relating to aging and gut physiology as well as spaceflight [21,111]. Further integrative study remains needed however for delineating those biomarkers that may best recover relevant network dynamics concerning cellular pathways, gut physiology, aging and spaceflight.

Experimental metabolomic studies in spaceflight mouse models have additionally identified significant alterations in liver metabolism, glutathione pathways, glutamate metabolism, inflammatory signaling, and lipid-associated oxidative stress responses following spaceflight exposure [112]. These findings support the hypothesis that spaceflight-associated oxidative stress perturbs interconnected metabolic and redox-regulatory systems across host tissues and microbiome-associated physiological environments. Additional studies have further implicated peroxisomal signaling and redox-regulatory pathways in oxidative stress responses associated with altered cellular maintenance and apoptosis-related signaling. Collectively, these observations support the view that oxidative stress influences a broad network of physiological processes extending beyond local tissues and organ systems.

Peroxisomes are redox-regulatory organelles that contribute to metabolic homeostasis, reactive oxygen species (ROS) signaling, and oxidative stress responses within eukaryotic cells [113]. Like mitochondria, peroxisomes are highly abundant in epithelial gut tissues and may participate in host–microbiome communication through regulation of metabolic and oxidative signaling pathways. Experimental studies in *Drosophila* gut models further suggest that interactions among peroxisomes, mitochondria, metabolites, and microbial communities may influence ROS regulation, oxidative stress accumulation, and cell death pathways within host tissues [114]. Using transcriptomic network analyses, Blaber et al. identified oxidative stress-associated pathways linked to altered cellular quiescence, autophagy, ubiquitin-mediated proteolysis, and apoptosis-related signaling during spaceflight, supporting the hypothesis that oxidative stress perturbs interconnected metabolic and cellular maintenance systems across host physiology [112].

**Figure 1:**
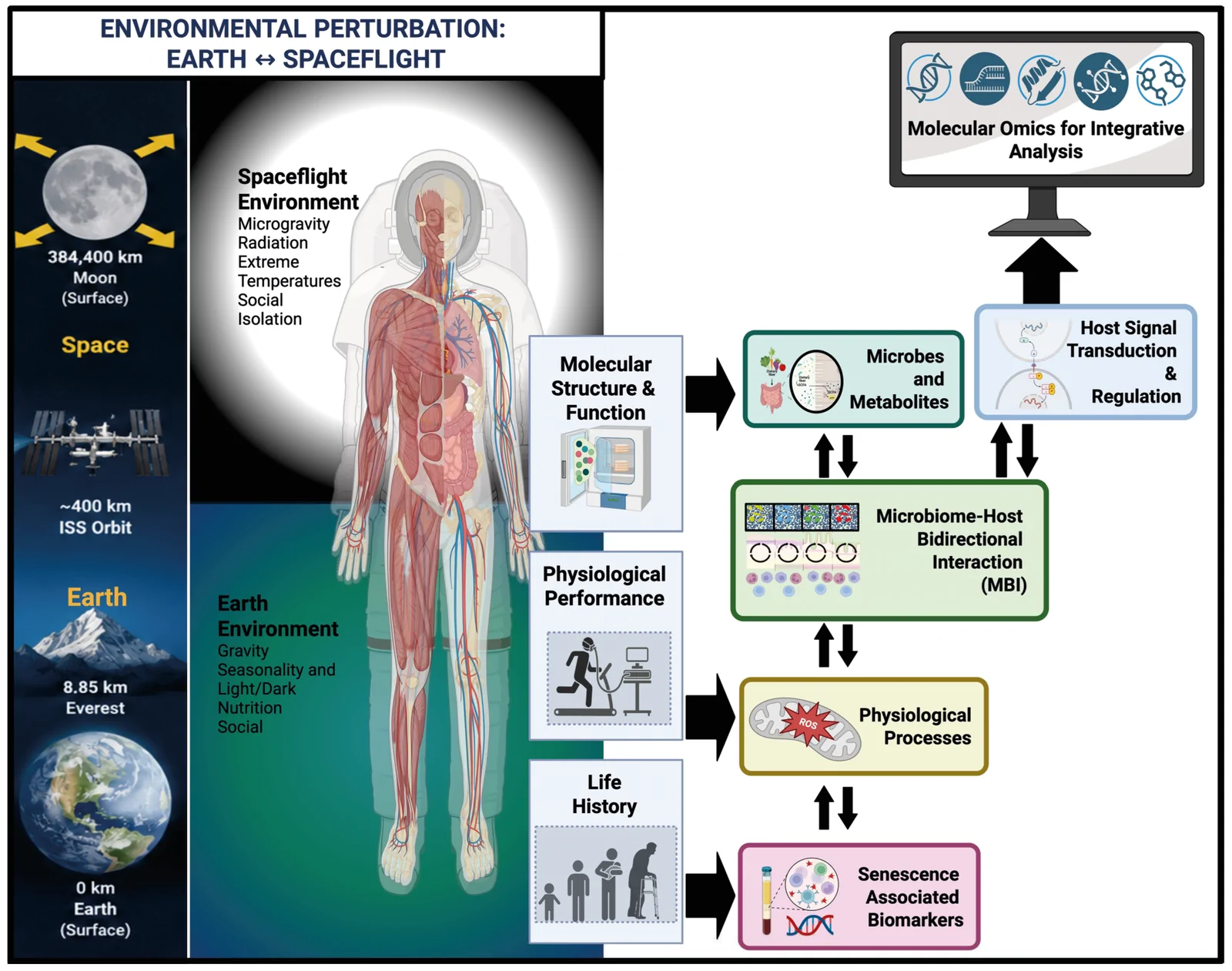
Analytical schema of how environment and life history can be investigated with respect to individual organisms. A central framework within this schema is molecule-mediated bidirectional interactions (MBIs). The figure is intended as an organizational model for evaluating molecular structure, molecular function, physiological performance, and biomarkers, as well as the role of molecular omics to achieve an integrative analysis. Figure partially created with BioRender.com. Naney, C. (2026) https://www.biorender.com/.

Figure 1 provides an organizational mode for how different environment conditions and the many different attributes of an organism can be investigated with the use of the MBI framework and molecular omics technologies.

## 4. Microbiome Dysbiosis, Infections, and Interventions for Promoting Healthier Gut Microbiomes and Decreasing Oxidative Stress

Recent investigations have increasingly implicated mitochondrial dysfunction, oxidative stress accumulation, and microbiome dysbiosis as interconnected components of aging and spaceflight-associated physiological decline [11,109]. Prolonged spaceflight and to a lesser extent simulated spaceflight exposure have been associated with accelerated aging-like phenotypes involving altered mitochondrial function, inflammatory signaling, and disruption of host–microbiome metabolic interactions. There is also a form of historical hysteresis in the advance of simulated spaceflight research. Many ground-based analogs have been developed before long-duration spaceflight datasets first became available, so their design reflects the prevailing understanding at that time of the most important physical perturbations associated with spaceflight, particularly altered gravity. As evidence of long-duration real spaceflight missions and secondary multi-omics analyses has arisen, it has become increasingly evident that real spaceflight represents a far more complex exposure landscape. This complex exposure environment includes a continuum of altered gravity, ionizing radiation, and confinement, and other factors. Consequently, modern simulated spaceflight studies increasingly minimize secondary mechanical artifacts, such as fluid shear generated in rotating devices, so that biological responses more closely reflect the intended effects of altered gravity rather than unintended mechanical stimulation [6,11,30,91,115,116].

These observations are broadly consistent with the mitochondrial theory of aging and related models proposing that progressive disruption of cellular energetics and redox homeostasis contributes to physiological decline across tissues and organ systems [16,117]. Consequently, growing attention has focused on interventions targeting oxidative stress regulation, mitochondrial maintenance, inflammation, and microbiome-associated metabolic pathways as potential countermeasures for aging-related dysfunction in both Earth-based and spaceflight environments.

Barrila et al. demonstrate a 3-D cellular model for host–microbiome interactions for comparing in vivo *Salmonella* Typhimurium infection on Earth, using a Low Shear Modeled Microgravity (LSMMG) culture in simulated microgravity, as an analogue for spaceflight exposure. *Salmonella* Typhimurium is the most thoroughly characterized microbial pathogen in spaceflight [27]. To prepare the bacteria for infection of human gut cells, they were first “passed through” the GI tissues of a model species, in this case a strain of *M. musculus*. Using the 3-D model, Barrila et al. detected epigenomic stress signals in the host genome and hypervirulence phenotypes in the wildtype strain of *Salmonella* Typhimurium, indicating a simultaneous increase in bacterial infectivity and host susceptibility in microgravity. The hypervirulence phenotype in wildtype *Salmonella* Typhimurium is associated with the z*irT* gene. When expressed, this gene assists bacterial attachment to the host oral cavity and is shed in mouse fecal material. It is also associated with the TET3 stress-response regulator in host physiology. In addition, the same study effects were even more pronounced in actual spaceflight compared to simulated spaceflight.

Studies of the biological and chronological age and the abundance of gut microbiome bacteria grouped by taxa have reported upon associations for specific bacterial lineages and networks [118]. Microbiome taxonomic abundance data generated using MetaPhlAn from the SpaceX Inspiration4 mission were available for preliminary hypothesis generation in this literature review [10]. The dataset includes microbiome samples collected from multiple host body sites at several time points before, during, and after spaceflight. Our own initial navigation of the dataset reveals bacterial taxa to change in abundance across crew members over the mission timeline, where bacterial taxa can vary in terms of converging or diverging in abundance over the mission timeline. Communities of interacting bacteria may also be evident. Maffei et al. identified a frailty-associated coabundance module comprising *Eggerthella*, *Coprobacillus*, and *Lachnospiraceae* taxa [118].

Cross-referencing Inspiration4 stool microbiome taxa with the Genome Taxonomy Database detected *Eggerthella* and *Coprobacillus*, although sample sizes were insufficient for statistical comparison. The analysis also identified nine enumerated novel species within the *Collinsella* lineage. Across sampled time points, these taxa showed declining median abundance and reduced inter-individual variability, suggesting convergence toward lower abundance across crew members during the mission and recovery period [116]. Together, these findings suggest a coordinated response within the *Collinsella* lineage and warrant further investigation to determine whether these shifts represent reproducible features of host–microbiome acclimation to spaceflight. Our further exploration of this database indicates that some *Bacteroides* species exhibit changes in abundance to conditions of spaceflight in comparison to other *Bacteroides* species having notably altered levels of abundance during post-flight recovery.

Due to inherent limitations in statistical power for small crew short-duration spaceflight missions, Inspiration4 observations should be interpreted as exploratory rather than confirmatory [9]. Nevertheless, the longitudinal structure of the dataset provides an opportunity to identify candidate microbial taxa associated with physiological adaptation to spaceflight and to generate testable hypotheses for future studies involving larger cohorts. Collectively, these observations suggest that microbiome adaptation to spaceflight is not solely characterized by directional abundance changes. Instead, taxa exhibited distinct response modes, including stability, coordinated abundance shifts, and transient convergence–divergence dynamics, consistent with the hypothesis that spaceflight-associated environmental pressures may restructure both microbial composition and inter-individual variability. Interpretation of the Inspiration4 microbiome data should also consider the heterogeneity of the crew. The astronauts differed in chronological age, sex, baseline physiology, and pre-flight microbiome composition. These sources of individual variation are important because microbiome abundance patterns and oxidative stress responses may be perturbed differently across individuals exposed to the same extreme environment. This makes Inspiration4 especially useful for preliminary hypothesis generation, but insufficient for confirmatory inference without larger cohorts and matched host physiological measurements [6].

Although not yet evaluated in spaceflight contexts, on Earth, a caloric study by Collino et al. focused on caloric restriction and insulin-like signaling upon all six of the standard mice strains commonly used in spaceflight studies. This work investigated potential increases in the production of new mitochondria in cells, and how this relates to the insulin pathway IIS, a “potent” regulator of aging [6]. In this study, Collino et al. further determined that caloric restriction interacted with some of the genes in their model, providing a protective effect on oxidative stress which was to be expected based on earlier findings from Lane [68,119]. For example, one of the putative genes was *Gstm6* (GO: 0004364), which is involved in glutathione metabolism, as a protective effect against oxidative stress in the liver of mice. This is one of a growing number of results supporting how caloric restriction increases longevity in mammals through various pathways [68,88,111,119,120,121,122,123,124,125]. Despite the caloric restriction of intervention findings on Earth, achieving the same longevity advantage in spaceflight may be more challenging. Spaceflight has a range of debilitating impacts on the body including decay in muscle mass, loss of bone density, and decline of cardiovascular function. Nonetheless, with oxidative stress being a key outcome in spaceflight, future studies in caloric restriction may represent a promising approach toward intervention.

## 5. Conclusions and Future Perspective

Evolutionary and physiological models of aging consistently describe senescence as a progressive decline in organismal function associated with increased mortality risk and deterioration of somatic maintenance systems. John Maynard Smith, Peter Medawar, and Michael R. Rose each have framed aging in terms of physiological decline occurring across life history as the force of natural selection weakens with age [66,126]. Because natural selection primarily acts upon survival and reproductive success, maintenance of somatic tissues is shaped by evolutionary trade-offs between energetic investment, reproduction, and long-term physiological stability [127]. Consequently, rates and mechanisms of senescence differ across tissues, organ systems, and species depending on genetic architecture, environmental exposure, and stochastic biological variation [128]. Understanding how spaceflight-associated stressors, including microgravity and cosmic radiation, interact with overall physiology and these conserved aging mechanisms remains an important challenge for future studies of human physiology in extraterrestrial environments.

The present review has therefore focused on the interconnected roles of mitochondria and microbiomes in aging and spaceflight physiology. Mitochondria are conserved cellular structures found in almost all multicellular life and have increasingly been proposed as central regulators of spaceflight-associated physiological stress and aging-related functional decline through their roles in cellular energetics, redox regulation, and oxidative stress signaling [11,17,72,129]. Parallel findings have additionally implicated microbiome dysbiosis, altered inflammatory signaling, and disruption of host–microbe metabolic interactions in both aging and prolonged spaceflight exposure. Collectively, these observations support the need for future integrative investigations examining how mitochondrial function, oxidative stress physiology, and microbiome dynamics interact across tissues, and physiologic systems (such as the immune system and endocrine system) in response to spaceflight-associated environmental stressors [4,11,115].

An example of how physiologic change and the potential for intervention relate to spaceflight and evolutionary mechanisms concerns spaceflight’s effects on the heart. Vernikos and Schneider have reported on magnetic resonance imaging measurements showing how the actual size of the heart decreases in spaceflight by 14%, which in turn compromises myocardial function. The change in size results in shear stress. The study authors report a similar decrease in heart size in patients undergoing long bed rest studies. In response, Vernikos and Schneider propose daily exercise as an intervention to potentially prevent shear stress [115]. From an evolutionary biology perspective, the Medawar, Williams, and Hamilton theory of aging rests upon population genetics and natural selection, and has been based on experimental evolution and exact, quantitative modeling [59,130,131]. These experimental evolutionary studies have investigated, for instance, hormetic responses. Hormetic responses could be correspondingly investigated using existing longitudinal spaceflight datasets. If hormetic responses contribute to host adaptation during spaceflight, these responses would be expected to arise through the conserved sensing and signaling mechanisms discussed throughout this review, including underlying host–microbiome dynamics. Collectively, hormesis includes all of the acclimatized responses that can protect a living individual under localized stress. Mechanistically, this approach is also expected to be associated with ROS/RONS. Spaceflight perturbs environmental conditions that are sensed through conserved signaling pathways. One consequence of these perturbations is altered cellular redox homeostasis involving ROS/RONS signaling, which has been implicated in adaptive physiological responses across multiple model organisms.

Beyond presenting an explanatory basis for how both physiologic state and evolutionary mechanisms account for physiologic change and potential intervention regarding spaceflight, a further opportunity for developing knowledge may reside with experimental evolutionary studies, including concerning aging and life-history tradeoffs across generations [132,133,134,135]. These experimental studies have enabled observation of age-associated changes in allele frequencies, stress resistance, and phenotypic traits across controlled evolutionary timescales. Expanding EE and LTEE approaches to include both Earth-based and spaceflight environments may further clarify how space-associated stressors perturb mitochondrial function, oxidative stress regulation, and expression of stress-response pathways and phenotypes [136]. Experimental evolution studies have additionally been used to investigate microbial adaptation to microgravity conditions [137]. However, most current investigations remain focused on single-species systems [138]. Future studies incorporating synthetic human gut microbiomes and multispecies host–microbe models in spaceflight environments may provide deeper insight into how complex metabolic and evolutionary systems respond to prolonged extraterrestrial exposure [111,139].

Understanding the physiological effects of spaceflight is inherently an interdisciplinary challenge requiring integration of evolutionary biology, metabolism, microbiology, genomics, systems biology, and aerospace medicine. As human spaceflight expands toward longer-duration missions and extraterrestrial habitation, continued investigation of mitochondrial function, oxidative stress physiology, and host–microbiome interactions will likely become increasingly important for monitoring physiological stress and developing targeted countermeasures [140]. Future advances in diagnostics, computational biology, and therapeutic intervention may improve the ability to preserve metabolic stability, immune function, and tissue maintenance in astronauts exposed to prolonged spaceflight environments.

## Figures and Tables

**Table 1:** Distribution of literature by experimental context and aging-relevant characteristics. Each count is based on the number of literature articles in the bibliography by category of study and experimental conditions. The Aging in Space column includes exposure to microgravity related to real spaceflight, simulated or computationally modelled microgravity, and secondary spaceflight data, including from other ground-analog spaceflight studies *.

Study Scope	Real Spaceflight (*n* = 13)	Secondary Analyses of Real Spaceflight (*n* = 32)	Simulated Spaceflight with Ground Analogs (*n* = 10)	On Earth Without Spaceflight Including Basal, Vivarium, and Ground Controls (*n* = 80)
Human	8	14	6	20
Mouse	3	9	4	8
Drosophila	0	2	1	7
Multiple Species	0	7	1	15
Microbiome	8	4	5	20
Mitochondria	0	4	1	18
Oxidative Stress	1	7	1	20
Multi-omics	2	4	1	2
Reviews	0	14	0	25
Methods, Standards, Resources	0	8	0	0
Perspectives	0	0	1	6

* Some of the counting represents assignments of individual articles to multiple scopes of study within each column category.

## Data Availability

No new data were created or analyzed in this study. Data sharing is not applicable to this article.

## Supplementary Materials

The following supporting information can be downloaded at: https://www.mdpi.com/article/10.3390/ijms27177644/s1.

## Abbreviations

The following abbreviations are used in this manuscript:

ATP	Adenosine Triphosphate
GI	Gastrointestinal
EE	Experimental Evolution
ISS	International Space Station
LTEE	Long-term Experimental Evolution
MBI	Molecules-mediated Bidirectional Interactions
OXPHOS	Oxidative Phosphorylation
RONS	Reactive Oxygen and Nitrogen Species
ROS	Reactive Oxygen Species
